# Chitosan Grafted Poly (Ethylene Glycol) Methyl Ether Acrylate Particulate Hydrogels for Drug Delivery Applications

**DOI:** 10.3390/gels8080494

**Published:** 2022-08-09

**Authors:** Corina-Lenuța Logigan, Christelle Delaite, Crina-Elena Tiron, Cristian Peptu, Marcel Popa, Cătălina Anișoara Peptu

**Affiliations:** 1 Department of Natural and Synthetic Polymers, Faculty of Chemical Engineering and Environmental Protection “Cristofor Simionescu”, “Gheorghe Asachi” Technical University of Iași, 700483 Iași, Romania; 2 Laboratory of Photochemistry and Macromolecular Engineering, Institute J.B. Donnet, University of Haute Alsace, Mulhouse, Street des Frères Lumière, F-68093 Mulhouse, France; 3 Regional Institute of Oncology, General Henri Mathias Berthelot Street, 2–4, 700483 Iași, Romania; 4 “Petru Poni” Institute of Macromolecular Chemistry, Aleea Grigore Ghica Voda, 41A, 700487 Iași, Romania; 5 Faculty of Medical Dentistry, “Apollonia” University of Iasi, Pacurari Street, 11, Iasi 6600, Romania Muzicii Street, No. 2, 700511 Iași, Romania; 6 Academy of Romanian Scientists, Splaiul Independentei Street, No 54, 050094 Bucharest, Romania

**Keywords:** chitosan particulate hydrogels, poly (ethylene glycol) methyl ether acrylate, double crosslinking, levofloxacin, biomedical applications

## Abstract

Chitosan (CS) crosslinking has been thoroughly investigated, but the chemical reactions leading to submicronic hydrogel formulations pose problems due to various physical/chemical interactions that limit chitosan processability. The current study employs the chemical modification of chitosan by Michael addition of poly (ethylene glycol) methyl ether acrylate (PEGA) to the amine groups to further prepare chitosan particulate hydrogels (CPH). Thus, modified CS is subjected to a double crosslinking, ionic and covalent, in water/oil emulsion. The studied process parameters are polymer concentration, stirring speed, and quantity of ionic crosslinker. The CPH were structurally and morphologically characterized through infrared spectroscopy, scanning electron microscopy, light scattering granulometry, and zeta potential, showing that modified CS allows better control of dimensional properties and morphology as compared with neat CS. Swelling properties were studied in acidic and neutral pH conditions, showing that pH-dependent behavior was maintained after grafting and double crosslinking. The applicability of the prepared materials was further tested for drug loading and in vitro delivery of levofloxacin (LEV), showing excellent capacity. CPH were found to be cyto- and hemocompatible demonstrating their potential for effective use as a controlled release system for different biomedical applications.

## 1. Introduction

The nanotechnology field is dedicated to designing and producing materials with nanometric dimensions by using different devices, structures, and systems that are inspired constantly by nature. The rapid progress of nanotechnology involved the development of new materials with different proprieties, such as biodegradability, biocompatibility, versatility, stimuli-sensitive properties, and the ability to deliver therapeutic biomolecules (drugs, proteins, genes, and photosensitizer) to a target in a controlled manner. Nanotechnology is already used in biomedicine applications through the use of liposomes, nanoparticles, and nanotubes, which are currently interesting and promising alternatives. Moreover, the chemical functionalization of natural/synthetic polymer nanomaterials offers a variety of opportunities such as tolerability of the polymer proprieties such as solubility, bioadhesivity, antibacterial properties, and antifungal activity [1].

Advanced nanomedicine systems require the combination of targeted delivery, imaging, and therapeutics in a single nanocarrier system. Hydrogel nanoparticles are promising nanomaterials due to advantages such as targeting, decreasing drug dose, the possibility of maintaining a desired therapeutic concentration, and low toxicity effects [2]. Moreover, a hydrogel is a polymer three-dimensional network, capable of capturing large amounts of water or active principles due to its hydrophilic structure, presenting excellent properties such as a porous structure, sensitivity to different environmental factors (pH, temperature, light), biocompatibility, and the similarity of their physical properties to natural tissue, which led to its use as support material in tissue engineering [3,4,5,6,7,8].

Chitosan (CS) is a cationic polysaccharide obtained via deacetylation and partial depolymerization of chitin under alkaline conditions or enzymatic hydrolysis, and is the second most common natural polysaccharide after cellulose”.

Although chitin is insoluble in most organic solvents, CS is soluble in dilute acidic solutions with pH < 6 due to the presence of primary amino groups (with pKa = 6.3) which become protonated. An increase in pH above 6, leads to the deprotonation of amino groups and CS loses its soluble character [9]. Chitosan represents a natural polymer with outstanding properties such as bioadhesivity, biocompatibility, biodegradability, non-toxicity, bioavailability, etc., which justify its attractiveness, especially for bio-related applications [2,9]. Moreover, CS shows important biological activity, including antiviral, antifungal, and antibacterial properties; antihyperglycemic, hypocholesterolemic, and antitumoral effects; genetic material transfection; and wound healing promotion [2].

The highly reactive amine moieties may be employed in a wide range of physical/chemical reactions for CS modification to gain a specific effect. One example is ionotropic gelation through ionic interactions between chitosan’s amine groups and negatively charged crosslinking agents, which results in a simple and flexible materials preparation procedure [10].

The diversity and efficiency of chitosan-based micro/nano-particulate hydrogel systems for drug targeting and controlled drug release applications have been extensively recorded in the literature [9,11,12,13]. Thus, physical crosslinking using small molecular weight anionic crosslinkers, such as sodium tripolyphosphate (TPP), has been employed in many studies [14]. However, the crosslinking process is reversible, and a pH increase leads to fast destructuring of the hydrogels through dissociation of the TPP/CS complexes into free chitosan chains [15]. Such phenomena are creating problems for applications that involve high pH variations, e.g., the passage through the gastrointestinal tract. A solution to alleviate the problems associated with the reversibility of ionic crosslinking in chitosan-based systems employs a supplementary covalent crosslinking step [13,15,16,17]. Chitosan’s particular chemistry favors crosslinking processes using aldehydes which form imine bonds with the amine functions [18]. Chitosan double ionic and covalent crosslinking has rarely been performed [18,19].

CS nanoparticles prepared via emulsification and crosslinking using the amino group of CS and the aldehyde group of glutaraldehyde (GA) were first obtained and characterized by Ohya et al. The nanoparticles were used as a system for circulatory delivery of 5-fluorouracil [20]. Furthermore, as an anionic crosslinker for CS, TPP is widely used and has been extensively investigated for the preparation of micro/nanoparticles, hydrogels, and scaffold systems in biomedical applications. For increasing micro/nanoparticle systems stability, both types of crosslinkers (e.g., TPP and GA) are needed [21]. Few studies are mentioned in the literature concerning the preparation of nanoparticle system-based CS or modified CS via the double ionic/covalent crosslinking method [16,17,22,23,24,25]. Adrenalin-loaded gelatin/CS particles for application in ocular drug administration were prepared using a two-step cross-linking process performed in an emulsion-phase separation system by Peptu et. al., (2010) [17]. Another interesting study concerning the obtaining of nanoparticles-based CS and gelatin via double crosslinking for drug targeting was realized by Jatariu (Cadinoiu) et. al, (2012) [22]. For the first time, polymer magnetic nanoparticles based on the chitosan-maltose derivative and magnetite double crosslinking technique (ionic and covalent) in reverse emulsion for antitumor drug delivery were prepared by Alupei et al., (2016) [16].

Damour I. and Muti H., (2021) succeeded in obtaining stable CPH based on lecithin/CS conjugates via a dual ionic/covalent crosslinking method. They found that the dual ionic/covalent crosslinking improved nanoparticles’ drug loading/releasing properties [26].

On the other hand, the chemical modification of CS is a promising method for achieving desired polymer properties. CS solubility in acidic solutions, its polycationic character, and the presence of NH_2_ groups in its structure, allow a variety of chemical modification strategies leading to new CS derivatives with new physical or biological properties [27].

The aim of this work is the chemical modification of chitosan by Michael addition of poly (ethylene glycol) methyl ether acrylate (PEGA) to the amine groups to further prepare submicronic particulate hydrogels. In this investigation, the synthesis of new submicronic particulate hydrogels based on CS grafted with poly (ethylene glycol) methyl ether acrylate (CS-PEGA) through a two-step cross-linking technique (ionic followed by covalent), in a reverse emulsion, is described for the first time. CPH characterization was carried out in terms of structural, size, morphological, swelling, and drug loading/release properties which were controlled by varying the synthesis parameters (polymer concentration, dispersion speed, and amount of ionic cross-linking agent). In vitro tests were performed to demonstrate CPH capacity for drug loading and release, hemocompatibility, and cytotoxicity. The two-step cross-linking technique was revealed to be a valid method for the synthesis of nontoxic CPH-based materials.

## 2. Results and Discussion

The work presented herein is focused on the effect of CS modification with PEG-acrylate on polymer processability during the preparation of submicronic nanoparticulate hydrogel drug formulations. The use of PEGA to chemically modify CS may lead to the improvement of its solubility in water and diminish its pH-dependent water swelling character by adding hydrophilic segments to the CS linear chain. In principle, native chitosan may be used for emulsion double crosslinking using ionic gelation in the first step followed by network fixation via covalent crosslinking at the emulsion interface level in the second step. Amine groups of CS are involved in both crosslinking steps and partial grafting with PEG-acrylate may be the source of uncertainties concerning the double crosslinking process. Moreover, the pegylated chitosan will further affect pH-dependent water swelling and drug release properties.

### 2.1. Synthesis and Characterization of CS-PEGA

**Scheme 1:**
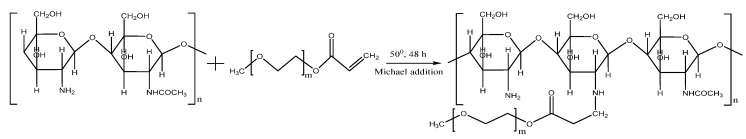
The proposed route for chitosan grafted poly (ethylene glycol) methyl ether acrylate synthesis.

The proposed pathway to synthesize CS-PEGA is a Michael addition reaction, which involves the addition of the acrylate PEGA end chains to the amino groups of CS, at 50 °C, as presented in Scheme 1.

**Figure 1:**
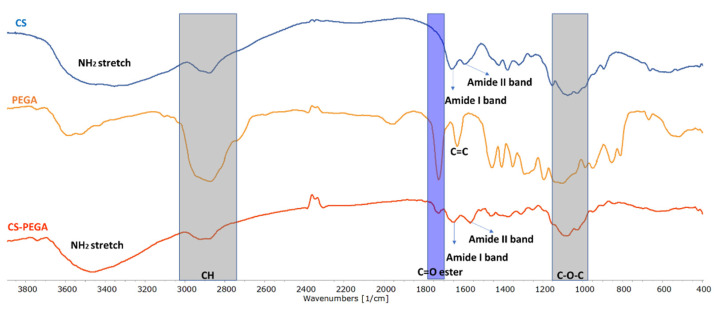
CS, PEGA, and CS-PEGA-FTIR spectra.

CS-PEGA structural characterization was performed via FTIR and ^1^H NMR aiming to have qualitative and quantitative proofs of CS grafting. Figure 1 illustrates the overlapped FTIR spectra of the starting precursors (CS and PEGA) and the synthesized polymer (CS-PEGA obtained using a molar ratio of NH_2_:PEGA = 1:0.5). The assigned characteristic bands from the obtained spectra are presented in Table S1.

FTIR CS spectra (Figure 1) showed absorption bands at 1076 cm^−1^ (C-O-C stretching vibration) [28], 1659 cm^−1^, 1423 cm^−1^, and 1378 cm^−1^ (NHAc units, amide I, NH_2_ bending, and amide III, respectively) [29]; and a strong peak at 3355 cm^−1^ attributed to the axial stretching vibration of (OH) overlapped with the (NH_2_) stretching band of CS molecules. The PEGA spectra show characteristic signals at 1631 cm^−1^ (C=C starching vibration), 1724 cm^−1^ (C=O asymmetrical and symmetrical stretching), and intense peaks at 2874 cm^−1^ (CH) and 3522 cm^−1^ (OH stretching vibration). The FTIR spectrum of CS-PEGA proves PEGA grafting onto CS through the appearance of the specific signal of (C=O) at 1733 cm^−1^, as highlighted.

**Figure 2:**
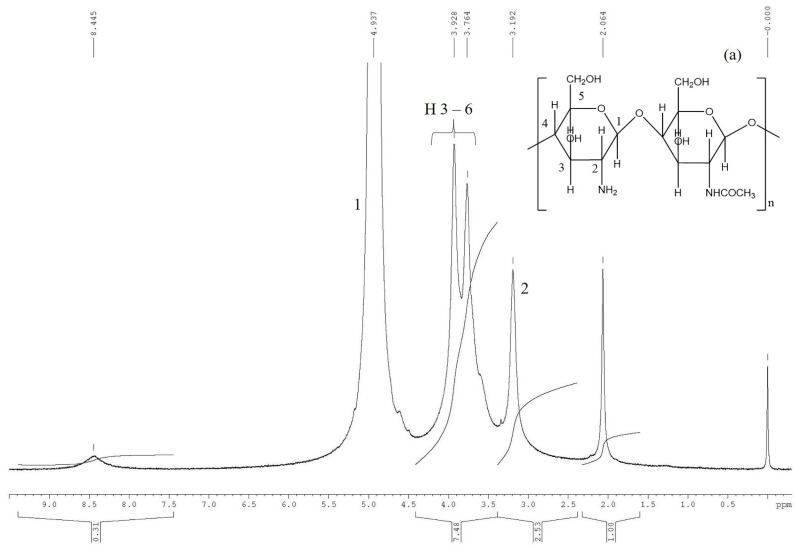

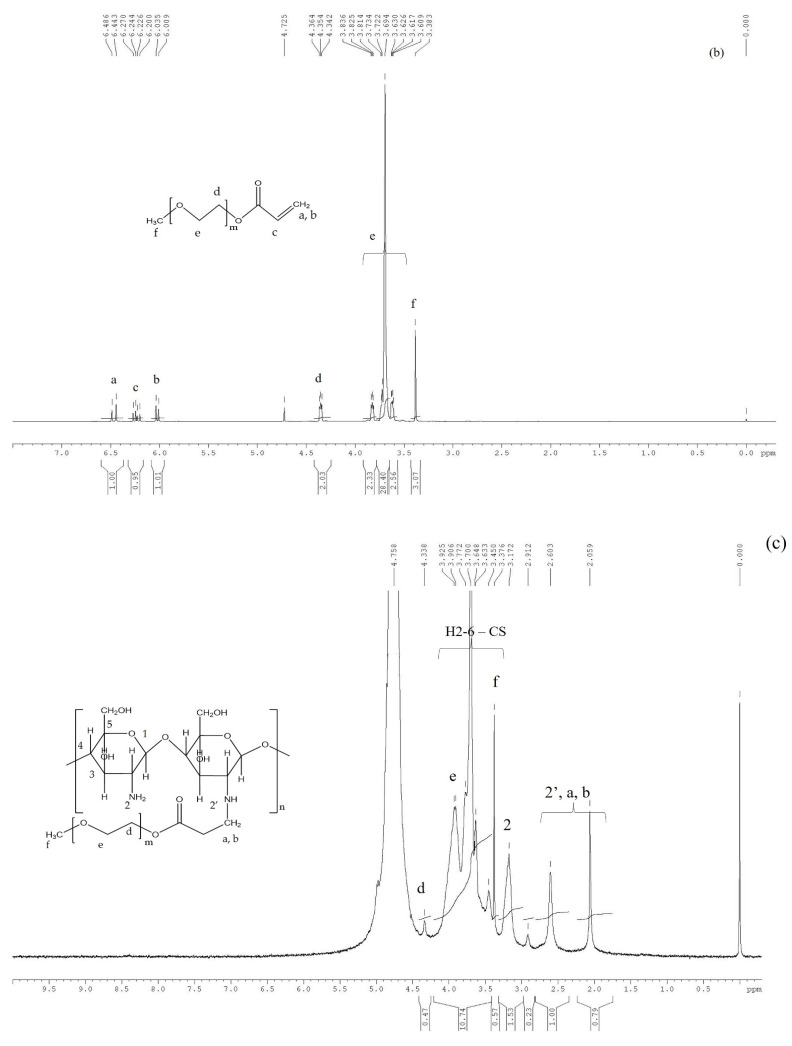
^1^H RMN spectra of (**a**) CS; (**b**) PEGA; (**c**) CS-PEGA.

^1^H NMR spectroscopy was employed to obtain more information regarding the chemical composition of the synthesized polymer. Figure 2 displays the ^1^H-NMR spectra of CS, PEGA, and CS-PEGA (molar ratio NH_2_:PEGA = 1:0.5 in PEGA synthesis feed).

The ^1^H NMR spectrum recorded for CS (Figure 2a) reveals the characteristic signal of methyl protons from the chitin residue, the resonance peaks of the acetylated group found at 1.90 ppm, and the peak corresponding to the H_2_ methine protons of the deacetylated groups at 3.01 ppm. H_3_, H_4_, H_5_, and H_6_ protons of the deacetylated and acetylated groups were observed in the 3.5–3.8 ppm range. Based on the spectrum, the hydrolysis degree of the CS was determined to be D = 88%.

The PEGA ^1^H NMR spectrum (Figure 2b) was recorded in D_2_O and the characteristic observed peaks were as follows: 6.44 ppm (CH_2_ = CHCOO), 6.24 ppm (CH_2_ = CHCOO), 6.005 ppm (CH_2_ = CHCOO), 4.35 ppm (–COOCH_2_–), 3.6–3.83 ppm (–COOCH_2_CH_2_–, –OCH_2_CH_2_–, –CH_2_CH_2_OCH_3_), and 3.38 ppm (–OCH_3_).

In the case of CS-PEGA, compared to CS, the peaks corresponding to the (–COOCH_2_CH_2_–) and (–NH-CH_2_CH_2_COO–) appear at 4.33 ppm and 2.60 ppm, respectively. A clear signal at 3.37 ppm corresponds to the (–OCH_3_) group of the PEGA structural unit. The signals of PEGA and in-chain methylene groups overlap with the signals of H_3_, H_4_, H_5,_ and H_6_ protons of CS. The CS-PEGA spectrum (Figure 2c) was similar to that obtained by Han et al., also confirming the successful synthesis of CS-PEGA [30].

The obtained spectrum was further used to calculate the CS substitution yield. Previously, the calculation of DS used the peak intensity of the PEG graft (–COOCH_2_–, 4.33 ppm) and (–CH_3_, 2.00 ppm) specific to chitin residues. Thus, DS calculated using Equation (1) was around 16%.

(1) $$\mathrm{DS}=\frac{3\cdot I_{4.3\;\mathrm{ppm}}\cdot \left(1-\mathrm{DD}\right)}{2\cdot I_{2.0\;\mathrm{ppm}}}$$

However, because of the influence of neighboring peaks, such calculations may be highly biased, and a different protocol was used for DS calculation taking into consideration only the CS backbone peaks:

(2) $$\mathrm{DS}=\frac{I_{2^{\prime }}}{I_{2}+I_{2^{\prime }}}100$$

where I_2_ is the integration of the peak found at 3.17 ppm, corresponding to the unmodified chitosan rings, and I_2′_ represents the integration of the modified chitosan, probably situated at 2.6 ppm. However, the NMR spectrum previously reported was slightly different [30] and in our case, the 2′ peak is overlapping with the peaks corresponding to the PEG methylene protons, a and b. Therefore, the 2′ integration value was indirectly obtained using the calculated CS hydrolysis degree, through Equation (3):

(3) $$I_{2^{\prime }}=\frac{I_{a^{\prime }}\mathrm{xD}}{3x\left(100-D\right)}-I_{2}$$

where D represents the calculated hydrolysis degree.

Finally, the obtained functionalization degree using Equation (2) was DS = 20%, much lower than that previously obtained by Han et al. In principle, higher substitution degrees may be reached (e.g., a 1:1 NH_2_:PEG molar ratio led to a DS of 51%) but our goal was to leave a certain amount of amine groups available while achieving complete water solubilization of CS-PEGA.

### 2.2. Preparation and Characterization of CPH

The proposed pathway to obtain CPH is a double crosslinking technique in reverse emulsion (*w*/*o*) [10,11,16,17,18,19]. The main problem related to the crosslinking procedure consists of the CS chemical modification impact on amine groups’ availability for further reactions. Furthermore, the improved water solubility of CS-PEGA should provide better preparation conditions for the particulate hydrogel’s formulation. The prepared systems were evaluated according to the following parameters: size and polydispersity, morphology, and zeta potential FTIR.

The selected method for particle preparation was the double crosslinking (ionic and covalent) technique in a water-in-oil emulsion, the advantage being the use of a lower amount of covalent crosslinker, which leads to a decrease in final product toxicity. The fundamental principle of the process is the formation of an interconnected/interpenetrated network in the first stage through the majority ionic crosslinking process, followed by the covalent crosslinking step. The experimental program concerning the preparation of CHP is presented in Table 1.

The parameters taken into consideration for the preparation process are CS-PEGA initial concentration, NH_2_/TPP molar ratio, and stirring speed in the emulsion formation phase. The initial water/oil phase ratio, surfactants concentration, and GA content in the organic phase were kept constant throughout the study.

Samples from A1 to A4 were obtained in the first experiments of the study and the molar ratio NH_2_/Na_5_P_3_O_10_ was varied at 1:0.5 (A1) and 1:1 (A2), the time of the ionic cross-linking process was varied at 20 min (A3) and 40 min (A4), while the water/oil phase ratio, surfactants concentration, and GA content in the organic phase were kept constant. SEM microphotographs showed a nonuniform spherical shape, which suggests that the amount of the ionic crosslinker (A1 and A2) or ionic reaction time (A3 and A4) were insufficient for their favorable formation. In addition, the formation of aggregates with average sizes of 2–3 μm and a fairly wide dimensional polydispersity, proves that they couldn’t be used in further investigations. After the first stages of optimization, which involved increasing the time of the ionic cross-linking/polymer ratio and molar ratio NH_2_/Na_5_P_3_O_10_, the prepared CPH presented a spherical shape, were individualized, and the diameter and polydispersity were much reduced compared to the first attempts.

The preparation yields (Table 1) revealed that the initial CS-PEGA amount was diminished during thorough purification procedures. Final yields were around 80% (samples A5, A6) at lower speeds and decreased at higher speeds to 76% (sample A7). Although yield variation is almost negligible, it could be considered that the centrifugation-washing cycles led to fractionation through the loss of the particle populations having the smallest size. Thus, after correlating the preparation yields with the preparation parameters, it may be observed that the highest yields are obtained at the lowest emulsification speeds because the emulsification conditions (stirring speed) favor the formation of larger size particles. The formation of larger size particles may lead to the encapsulation of CS-PEGA material with low network connectivity as compared with lower size particles. In principle, the polymers which do not benefit from crosslinking during the preparation process are eliminated during the purification step, contributing to lower yields. Thus, larger-sized particles may encapsulate free chains that are not eliminated by purification, increasing the final yields.

Particle size measurements confirm that preparations performed at lower emulsification speeds result in larger particle size. Furthermore, the initial concentration of the CS-PEGA affected the preparation yields. Thus, switching the initial concentration from 0.5% to 0.35% (wt) led to a decrease in the preparation yields from 78% to 56% together with a drastic reduction in particle average size from A8 to A9. On the other hand, increasing the polymer concentration to 0.75% (wt) also led to diminished yields associated with the loss of free polymer. Higher polymer density may affect the crosslinking processes, both ionic and covalent. Finally, the increase in TPP amount is correlated with a particle size reduction, from 562 nm (A8) to 141 nm (A10) and, consequently, with diminished yields from 78 to 59%.

CPH samples were also analyzed by zeta potential measurements and Table 1 summarizes the arithmetic average of zeta potentials. The analyzed samples were dispersed in distilled water at a mass concentration of 1.25 g/L and sonicated for 15 min to avoid agglomeration. In principle, a large negative or positive value of zeta potential for suspended particles in an aqueous medium indicates that the system is stable against flocculation or coagulation. As shown in Table 1, the obtained results for samples A8, A9, and A10 indicate that CPH have good stability in aqueous suspension. The measured zeta potential values are explained by the lower availability of the amine groups due to PEG grafting and covalent crosslinking.

#### 2.2.1. CPH Characterization

**Figure 3:**
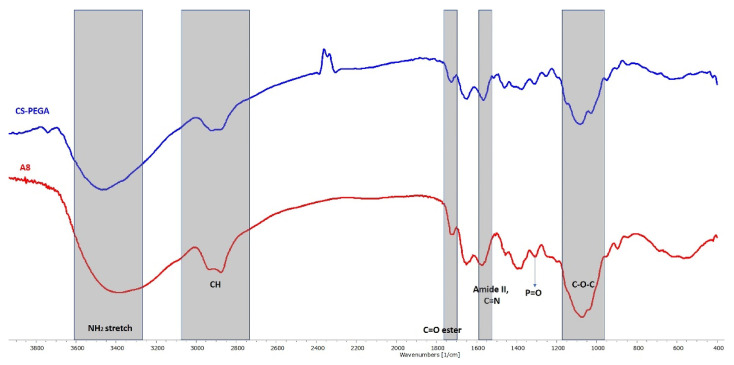
FTIR spectra of the modified chitosan (CS-PEGA) and chitosan particulate hydrogels (A8 sample).

To better understand the polymer network formation after the double crosslinking process, CPH were first investigated by FTIR spectroscopy. The recorded spectra revealed a similar profile for all CPH and confirmed the success of both crosslinking processes, thus only the FTIR spectra for sample A8 are displayed (Figure 3). Table S2 presents in detail the characteristic absorption bands. The first crosslinking process confirmed was the ionic one, the spectra revealing at 846 cm^−1^ the presence of a new bond formed between P_3_O_10_^5−^ anions of TPP and NH_3_^+^ cations from CS-PEGA. The characteristic absorption bands corresponding to the covalent crosslinking process, namely C=N, were observed at 1573 cm^−1^ [13,16,31,32,33,34].

**Figure 4:**
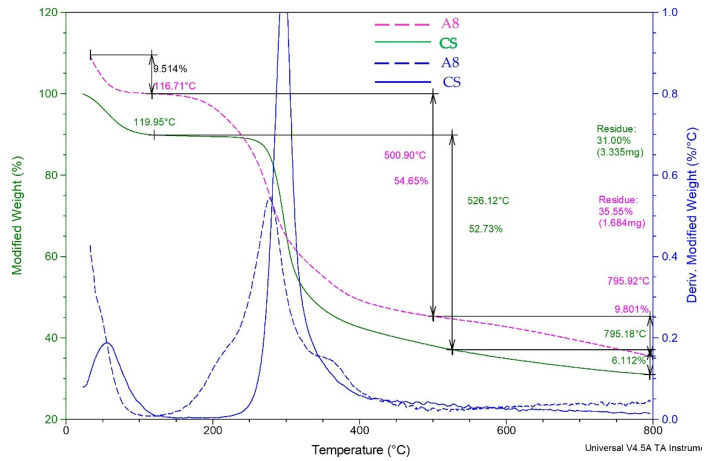
TGA analysis of chitosan (CS) and chitosan particulate hydrogels (A8 sample).

Furthermore, the CPH were also subjected to TGA analysis (Figure 4). The data results from the TGA analysis indicated that CPH decomposition proceeds in three steps as shown in Figure 4. TGA thermograms (Figure 4) showed a steady weight loss in the temperature range of 100–500 °C. The slight weight losses (9.514% and 10.13%) up to 116 °C, and 119 °C respectively, correspond to adsorbed water and moisture loss by the CS and CPH. The second decomposition step (A8—54.65%, CS—52.73%) in the temperature range of 116/119–500 °C is mainly due to the degradation of oligosaccharides and PEG. The main difference between CS and A8 samples is related to the early onset of A8 degradation due to the chemical modification in correlation with the native CS. Thus, the presence of covalent GA bridges is probably the reason for the decrease in thermal stability. Moreover, the A8 sample presents a distinct extra shoulder in DTG corresponding to the additional PEG, between 350 and 400 °C. Overall, thermal analysis confirms the structural composition of A8 concerning CS-PEGA content and covalent crosslinking. Supplementary TGA analysis performed on A9 and A10 samples (Figure S1, Supplementry Material) showed that different preparation conditions, such as concentration and TPP amount, do not significantly influence the TGA profile.

#### 2.2.2. CPH Morphology

**Figure 5:**
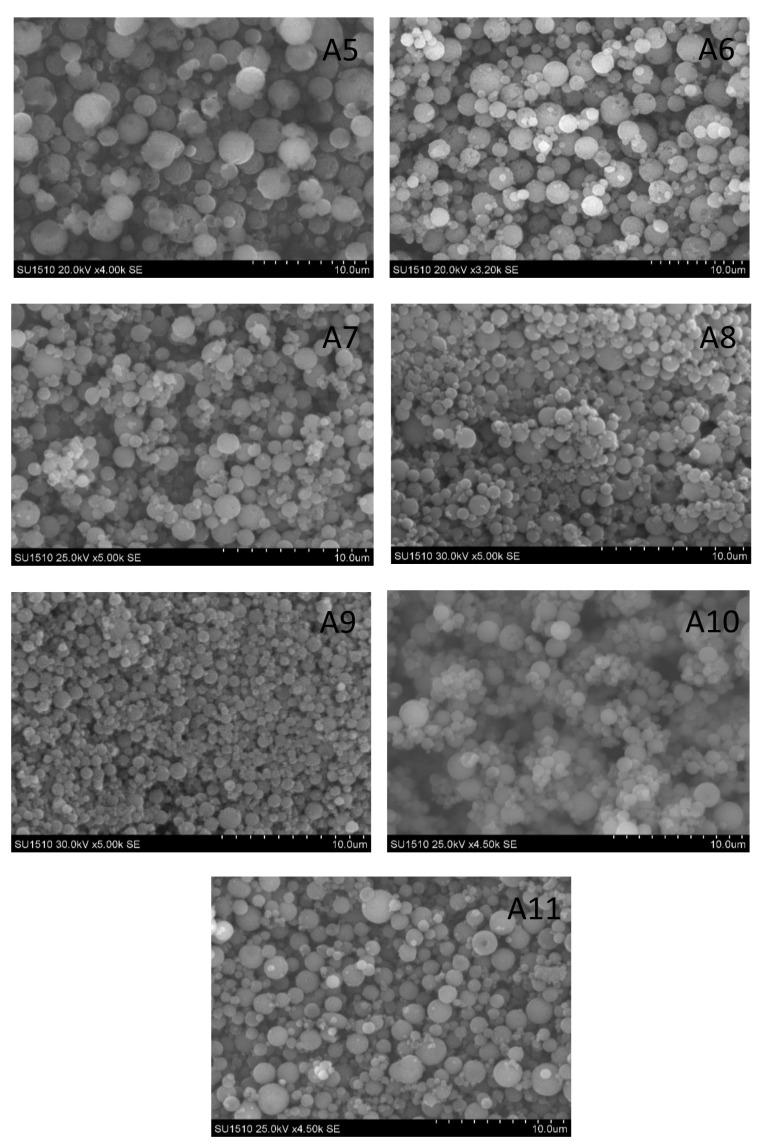
Micrographs SEM of CPH (magnification graphical bar length A5–A11:10 μm).

SEM technique was used to inspect the morphology of the prepared submicronic gel systems, Figure 5. Results indicate that they have a spherical shape, are well individualized, generally have a submicronic diameter, and reduced dimensional polydispersity. The adapted emulsion double crosslinking procedure allows the obtaining of clear particulate systems with relatively low dimensional dispersity, as observed from SEM micrographs. Furthermore, it may be remarked that particles are homogeneous, separated, and lack impurities resulting from surfactants, crosslinkers, or CS-PEGA residues from faulty emulsion crosslinking.

The influence of the preparation parameters is well reflected in the size of CPH, especially the lowering of the concentration of CS-PEGA, which produced particulate gels with the lowest dimensions. A visual comparison between the A8 and the A9 samples (Figure 5) clearly shows the dimensional decrease.

**Figure 6:**
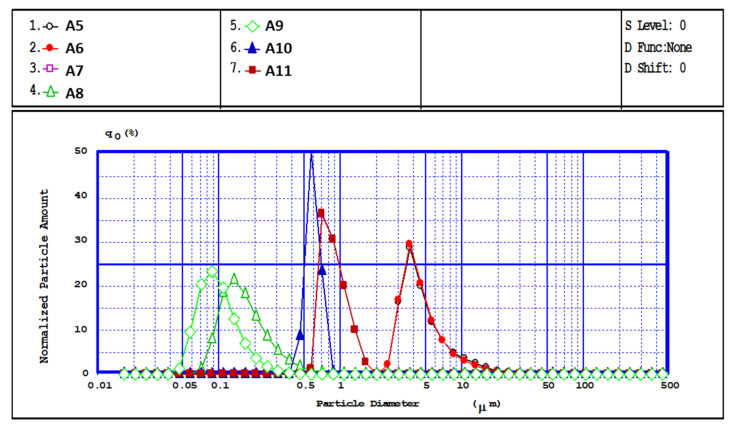
Granulometric distribution curves of CPH.

Furthermore, a more quantitative idea of the dimensional distribution of particle populations is obtained by dimensional analysis via laser diffraction (Figure 6), which provides the diameter and dimensional polydispersity of CPH. The analysis was carried out in acetone, known as a poor solvent for CS, and the measured average diameters were in good agreement with SEM observations. The experimental data confirmed that the particle size is dependent on the polymer solution concentration and stirring speed used during preparation. Thus, a higher stirring speed implies higher energy dissipated in the stirred emulsion, i.e., a higher energy transfer, which causes the emulsion to break into smaller droplets, resulting in nanometric particles. The granulometric distribution curves of the CPH obtained (determined by laser beam diffraction (LD)) have a unimodal allure and a diameter falling in the submicronic range, depending on the preparation conditions (Figure 6 and Table 1). Thus, for samples A5–A8, after increasing the stirring rate from 5000 to 15,000 rpm/min, it was noticed that particle size decreases due to better dispersion and formation of small droplets in the emulsion phase.

The influence of the concentration of CS-PEGA solution on particle size was also analyzed for three different polymer concentrations, namely 0.35%, 0.5%, and 0.75%, while the other parameters were kept constant (samples A 8, A9, A11, Table 1). The lowest polymer concentration led to particles with sizes in the nanometric range and an average diameter of 89 nm, and higher concentrations resulted in larger particles. Additionally, increasing the polymer solution concentration led to a considerable increase in the mean diameter and particle polydispersity, as well as a decrease in agglomeration tendency. The observed tendency is explained by the fact that higher concentrations lead to an increase in solution viscosity, and therefore to the formation of larger emulsion droplets, as presented by other studies [35,36].

Another studied parameter, the increased polymer/ionic crosslinker ratio (A8 vs. A10), caused a noticeable decrease in particle diameter, from 562 to 141 nm. Better availability of TPP may favor ionic gelation in smaller colloids in the early emulsification process. Furthermore, in the ionic crosslinking phase, due to the increase in the cohesion of the polymer matrix, the emulsion droplets should better maintain their colloidal stability. Covalent crosslinking improves and fixates the resulted particles which otherwise may suffer modifications in size.

Overall, considering the small size and dimensional uniformity of the prepared particulate hydrogels, the use of CS-PEGA in double crosslinking processes is an improvement on previous efforts. TPP ionotropic gelation alone leads to particle sizes of around 300 nm [14] while previous double crosslinking attempts on native or modified CS led to a minimum particle size of 500 nm. The current work shows that PEG grafting up to 20% does not affect the capacity of CS to undergo crosslinking reactions because of sterical effects and amine moieties coverage.

#### 2.2.3. Water Uptake

The swelling capacity was studied in two environments, which were closely related in terms of CPH drug loading and release potential. The acidic/basic standard buffer medium was chosen because, in general, the swelling behavior of CS hydrogels can be influenced/dependent on external triggers such as pH, ionic strength, the temperature of the environment, and the degree of cross-linking. Furthermore, in the case of CS hydrogels which are pH-dependent, the loading/release behavior of active principles is related to a higher swelling capacity in the acidic environment due to a significant increase in their volume which leads to an accelerated drug caption inside the network [36].

To better understand the interaction between CPH and the aqueous environment, swelling studies were performed in acidic (pH = 3.3) and respectively alkaline (pH = 7.4) mediums, using the gravimetric method. The water swelling behavior gives insights into doubly crosslinked CS-PEGA interactions with selected environments and its adequacy for the drug loading and release process.

**Figure 7:**
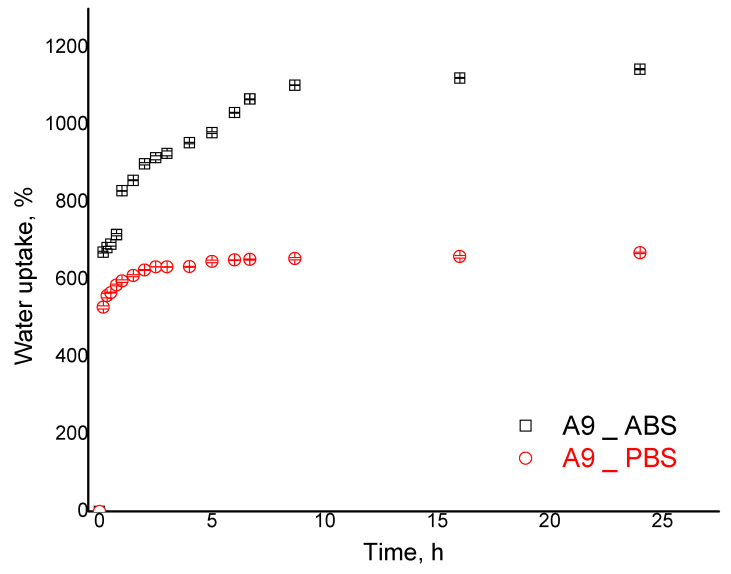
Chitosan particulate hydrogels (A9 sample) pH-dependent water uptake in pH = 3.3 (ABS) and pH = 7.4 (PBS).

The kinetic data are presented as an example for sample A9 (Figure 7). The results revealed two phases in the acidic environment: a fast increase up to 800% (sample A9) in the first 90 min, followed by a slower water uptake increase rate (90–480 min); the maximum value is reached after 24 h at 1144%. This behavior may reflect the double crosslinked nature of CPH. In the case of PBS, the maximum value of the swelling degree was smaller at 669%. Swelling kinetics presented in Figure 7 reveal that an equilibrium phase is established in both acidic and alkaline environments, showing good stability of the particulate hydrogel’s formulations. Although in the alkaline environment such behavior would be expected because of diminished electrostatic repulsions among polysaccharide chains, the stability in the acidic environment confirms successful covalent crosslinking.

The water uptake in both environments proceeded quickly, demonstrating good water–CS interactions, most likely favored by hydrophilic PEG grafts. However, the remaining free amine groups dictate the swelling behavior as acidic ABS determined significant water swelling. Therefore, we can infer that CS-PEGA provides a good prospect for nanoparticulate hydrogels by diminishing the large difference in CS behaviors in acidic and alkaline environments.

Furthermore, the water interactions of CS-PEGA nanoparticulate hydrogels are discussed according to the maximal water uptake reached in the equilibrium swelling phase (Table 2). Generally, water swelling of homogeneously crosslinked polymer networks is influenced by polymer–water interactions and the network’s capacity to expand its volume, influenced in turn by chain conformations, flexibility, and network crosslinking density. However, in the case of double crosslinked particulate hydrogels, the crosslinking is heterogeneous. GA covalent bridges are formed starting from the organic phase–water interface. In principle, because of its high reactivity with amine groups, GA diffusion into the water phase is limited and it can be considered that a layer on the surface of particles benefits from the covalent crosslinking while the inner polymer matrix remains mostly ionically crosslinked. The lack of crosslinking anisotropy further generates specific particle–water interactions. The outer layer of covalently crosslinked polymer is the key factor that determines the swelling behavior. By comparing the water uptake of samples A8 and A10 (Table 2), with an increasing amount of ionic crosslinker, it was noticed that there were insignificant differences between the acidic and alkaline environments. Such behavior is in opposition to the increasing network crosslinking density and also to the possible network dissociation due to the neutralization of the ionic bridges in an alkaline environment.

**Figure 8:**
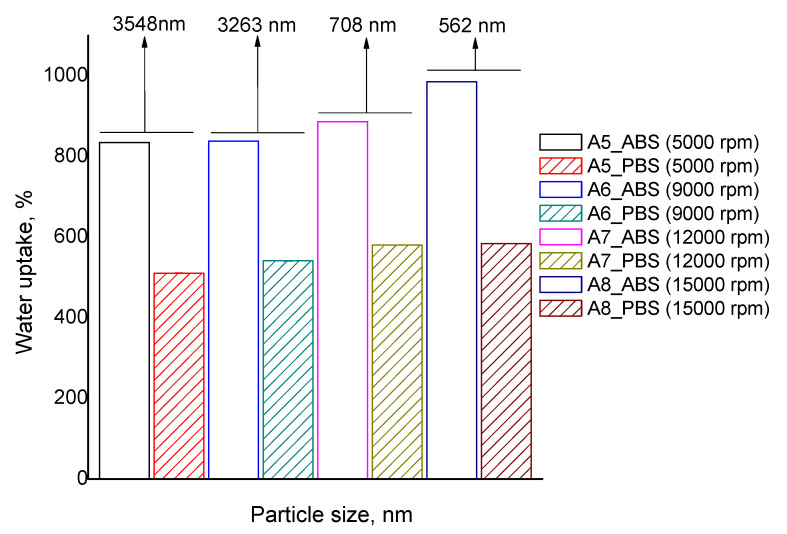
The influence of CPH size on the swelling process for CPH: A5 (5000 rpm); A6 (9000 rpm); A7 (12,000 rpm); A8 (15,000 rpm).

In principle, an increase in the surface of the particles is associated with a decrease in the particles’ average diameter. Taking this into consideration it can be inferred that particle size is influencing the swelling behavior. The evolution of water uptake according to particle size is presented in Figure 8.

The decrease in particle size of the considered samples is only due to a more energetic initial emulsion stirring and the other parameters influencing crosslinking, such as ionic/covalent crosslinkers and polymer concentration, were kept constant. The acidic swelling for particles with an average diameter of 3540 nm is around 835% (sample A5), and a decrease in particle size to 562 nm leads to higher swelling, up to 985% (sample A8). Furthermore, in the alkaline environment, the swelling increases from 511 to 585%. Thus, water uptake shows a clear dependency on the average diameter of particles in both acidic and alkaline environments, but the effect is more pronounced in ABS swelling.

**Figure 9:**
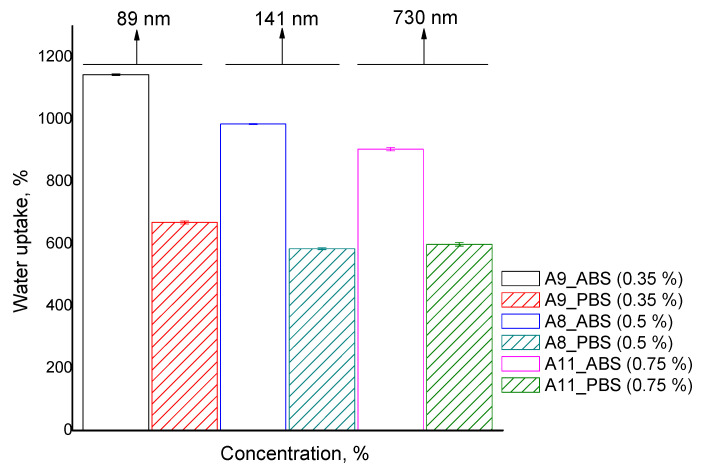
Polymer concentration influence on water uptake for CPH: A9 (0.35%); A8 (0.5%); A11 (0.75%).

The water uptake experiments were performed to investigate the dependence between the swelling degree and polymer concentration (Table 2 and Figure 9). The data presented in Figure 9 show the evolution of the swelling degree for three different concentrations and it is observed that increasing the CS-PEGA concentration in the emulsion feed diminishes the particle water uptake in both ABS and PBS. However, the swelling decrease is more obvious in an acidic environment, from 1175% (sample A9) to 900% (sample A11). This behavior is to be expected due to more dense polymer networks with a concentration increase.

Taking into consideration the significant differences observed in water uptake behavior in ABS and PBS, it can be affirmed that CS-PEGA particulate hydrogels have a pH-sensitive character. The effect is determined by the amine groups of the CS derivative which didn’t participate in the crosslinking process (both ionic and covalent). In an acidic environment, the amine groups are quaternized by passing into ammonium cations, and the electrostatic rejections between them increase the distance between network chains, and their meshes, leading to higher water uptake. The maximum swelling values are lower in a basic pH compared to an acidic pH, because the amine groups of the CS derivative are deprotonated, therefore, hydrogen bonds are formed in the newly formed polymer network.

The best swelling ratio was achieved by sample A9, obtained at a low polymer solution concentration (0.35%) and with the smallest particle size (89 nm). The higher water uptake for CPH obtained is probably due to the increased agglomeration tendency, which is mainly evident in sample A9, and a low cross-linking density of the polymer matrix.

#### 2.2.4. Drug Loading and Release

The ability of CPH to encapsulate active principles was analyzed using levofloxacin (LEV) as a model drug. LEV is a synthetic antibacterial drug of a broad-spectrum antibiotic belonging to the class of fluoroquinolones, generally used to treat antibacterial infections. LEV is a hydrophilic drug and therefore a suitable candidate for hydrogel particulate systems designed for the treatment of bacterial infections [35,37]. Taking into account the swelling results, which demonstrate that in acid pH the swelling ability of CPH is much higher than in weak alkaline conditions, it was decided to load the LEV in acid conditions ensuring a higher amount of loaded LEV. The LEV content in the supernatant was determined by UV-Vis spectrophotometry (at 287 nm wavelength) based on an established calibration curve. CPH were able to encapsulate between 0.48–0.75 mg drug/mg particles (Equation (6)). Therefore, the LEV loading efficiency values calculated with Equation (7) were between 47.0–76.0%, showing a high capacity of the CPH to support the biologically active compound. The amounts of encapsulated LEV in CPH and loading efficiencies are outlined in Table 3.

The values obtained at different time intervals showed that after 72 h the amount of encapsulated LEV ranged from 0.48–0.75 mg per mg of CPH. The kinetic data presented as an example for samples A8 and A9 (Figure S2), showed that LEV is loaded in two phases, a fast process during the first 10h, followed by a slower drug loading until 72 h. When comparing the drug loading profile with the swelling results, a similarity could be observed.

**Figure 10:**
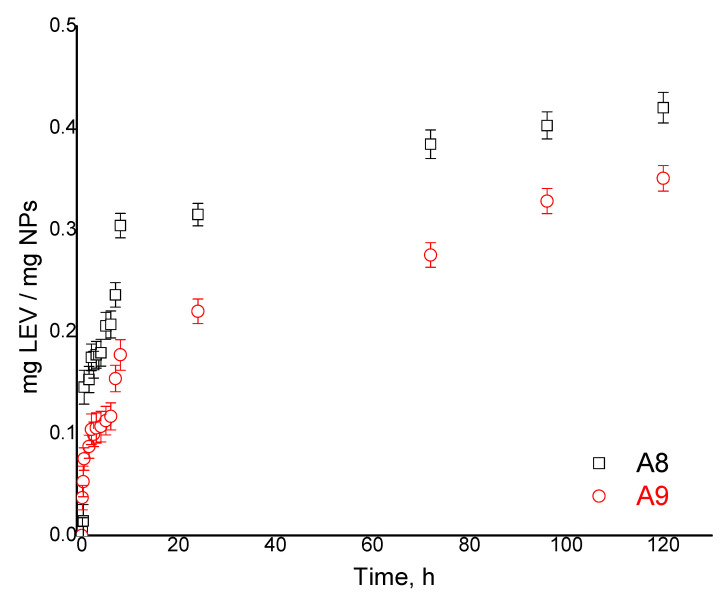
Chitosan particulate hydrogel in vitro LEV release.

For the LEV release study, pH = 7.4 was considered appropriate for a future systemic administration, acidic release conditions being used only for oral formulations. The LEV release capacity of CPH was analyzed by the diffusion method in phosphate buffer pH = 7.4 at 37 °C. The released LEV for all samples is presented in Table 4 and the kinetic data for two representative samples, A8 and A9, are displayed in Figure 10. One can observe a fast release phase, which is reached within the initial 10 h, followed by a slower phase (characterized by a slow release) until 120 h. The sustained release of LEV can be explained by the fact that the released drug was adsorbed in the CPH network due to the excellent hydrophilicity of the polymer network, which it seems is retained after the crosslinking procedures.

By analyzing the influence of preparation parameters on the release ability of the CPH, similar behavior as for drug loading was observed. The maximum released amount of LEV varied between 0.27 and 0.42 mg/mg CPH (Table 4). Furthermore, when calculating the efficiency of LEV release for all samples, values between 41 and 60% were obtained (Figure S3), the highest efficiency being in sample A8, which has also shown a high water uptake. The analysis of the release kinetics was achieved based on the Korsmeyer–Peppas mathematical model (Figure S4).

According to the literature [38,39,40,41], the drug release process is dependent on the method of obtaining the particulate systems, their dimensions, and the physical–chemical properties of the polymer support used. After analyzing the experimental kinetic data for the interval 0–480 min, it was possible to determine the diffusion exponent based on the equations listed in Table S3. As an example, Figure S4 shows the results obtained for samples A8 and A9. One can observe that for sample A8, the value of the exponent *n*, which characterized the release mechanism, is quite close to 0.5 (0.5 < *n* < 1.0), indicating a transport/release mechanism dominated by diffusion. Furthermore, it can be stated that sample A11 exhibits an abnormal, non-Fickian diffusion, the drug transport process through the polymer matrix being governed by both diffusion and swelling.

**Figure 11:**
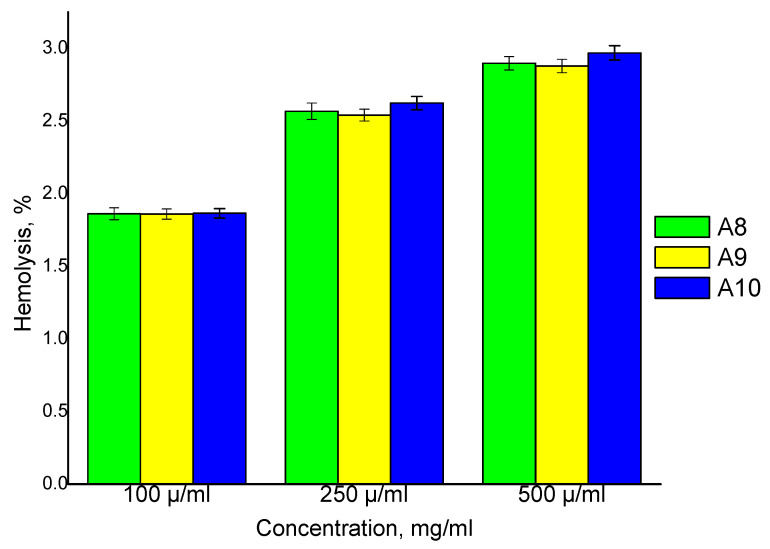
The influence of CPH concentration on the degree of hemolysis (samples A8, A9, A10).

The CPH hemolytic potential test is a mandatory requirement for blood-borne materials, since their interaction with blood components may lead to erythrocytes lysis. For this reason, the effects of the prepared CPH on blood were evaluated using a hemolysis assay. The results obtained from this study revealed that the degree of hemolysis increases with an increase in the CPH concentration (Figure 11). The degree of hemolysis for three different samples (A8, A9, A10) was analyzed and the results showed that there were no significant differences between them at the same concentration. It has been found that the prepared CPH shows very good compatibility with normal blood (<10% compared to the positive control) [41].

The viability of HDMVEC cells (Primary Dermal Microvascular Endothelial Cells) in 2D culture treated with nanoparticulated formulations (A9) was not affected by the presence of glutaraldehyde in the CPH structure (Figure S5).

#### 2.2.5. Cell Proliferation

For cell viability, a CellTiter-Blue^®^ Cell Viability Assay (Promega, Madison, WI, USA) was used. Cells were seeded into a 96-well flat-bottomed tissue culture plate at a density of 3000 cells/well and allowed to adhere to the plate by incubating at 37 °C under 5% CO_2_ overnight. Following cell attachment, the cells were incubated with 0.5 and 1% CPH for 72 h. After 72 h treatment periods, 50 μL of cell viability solution was added to each well and the plate was reincubated for 4 h before fluorescence recording using a multiplate microplate reader (FilterMax F5, Sunnyvale, CA, USA). As one can see from Figure S5, analyzed CPH does not affect cell viability according to statistical analysis. The statistical analysis of significance in differences was performed using a GraphPad Prism 7 Anova *t*-test.

CPH formulation-based CS-PEGA does not affect cell viability, thus confirming that they are cytocompatible and can be used as a drug delivery system.

## 3. Conclusions

This work demonstrates the suitability of CS-PEGA for the preparation of particulate hydrogel systems through double emulsion crosslinking. The relatively low substitution degree of chitosan with PEG-acrylate, about 20% as determined by ^1^H NMR, ensures sufficient availability of the remaining amino functions for further ionotropic gelation with TPP and covalent crosslinking with glutaraldehyde in water in oil emulsions. The outcome of the particle preparation procedure may be finely tuned by playing with process parameters such as water phase concentration, emulsification stirring speed, and amount of ionic crosslinker. According to SEM analysis, the CPH have a spherical shape, are homogeneous, separated, and lack impurities. The particle average diameter, measured by laser diffractometry, depends on specific preparation conditions and ranges from 3500 to 90 nm, in agreement with SEM estimations. Particularly, emulsification speed and water phase concentration significantly influence the CPH size and allow fine-tuning, according to the envisaged applications.

CPH behavior in two different aqueous media, acid (pH = 3.3) and basic (pH = 7.4,) was studied, demonstrating a higher water retention capacity for the acidic media, compared to basic media. Furthermore, the water uptake properties are significantly influenced by polymer concentration and ionic crosslinker amount employed in the preparation stage, and they are correlated with the particle size. The drug loading and release capacity of CPH, tested using levofloxacin, showed a good diffusional loading capacity, up to 0.75 mg of LEV per mg of CPH in an acidic environment. Drug rapid release in physiological pH was observed within the first 10 h, followed by a slower phase, characterized by a constant release up to 120 h. Drug release kinetics analysis demonstrated that the drug transport/release mechanism is predominantly diffusional.

CPH have also been tested by hemocompatibility analysis, the results showing a hemolysis percentage of less than 3%, indicating that they are hemocompatible. Cytocompatiblity studies demonstrated that CPH formulations based on CS-PEGA do not affect cell viability, thus confirming their low cytotoxicity and recommending them as pH-dependent drug delivery systems. Further experiments will consider the antibacterial potential of the levofloxacin-loaded chitosan particulate hydrogels.

## 4. Materials and Methods

### 4.1. Materials

Chitosan (CS, low molecular weight, 88% hydrolysis degree); Poly (ethylene glycol) methyl ether acrylate (PEGA, Mn = 480); Acetic acid (99%); Glutaraldehyde (GA) (25% aqueous solution); Sodium tripolyphosphate (TPP); levofloxacin (LEV); Tween 80; Span 80; acetate and phosphate-buffered saline (PBS) were purchased from Sigma-Aldrich, Saint Louis, MO, USA; MCF-10A cells were purchased from American Type Culture Collection, Manassas, VA, USA; DMEM/F12 supplemented with 5% horse serum (Sigma Aldrich, Saint Louis, MO, USA); 20 ng/mL EGF (Sigma Aldrich, Saint Louis, MO, USA); HDMVEC (Primary Dermal Microvascular Endothelial Cells) are from ATCC, Manassas, VA, USA; Milli-Q ultrapure distilled water (Merck, Rahway, NJ, USA). All other reagents used in this study were of analytical grade purity and were used without further purification. The human blood samples used were freshly obtained from one healthy nonsmoker volunteer.

### 4.2. Methods

#### 4.2.1. Synthesis of Chitosan Grafted Poly (Ethylene Glycol) Methyl Ether Acrylate

Chemically modified CS with PEGA via Michael addition reaction was performed according to a protocol reported by Han et al. [30]. Briefly, CS (2.0 g) was dissolved in 100 mL CH_3_COOH solution 1% (*w*/*w*) and added to a 250 mL reaction flask equipped with a reflux condenser. Next, the reaction solution was immersed in a water bath and heated at 50 °C, purged with nitrogen, and stirred for 30 min. Subsequently, PEGA was added dropwise (molar ratio NH_2_:PEG = 1:0.5). After 48 h the reaction was complete. The reaction mixture pH was raised from 3.4 to 8 with NaHCO_3_ saturated solution. Subsequently, the polymer solution was filtered via centrifugation and precipitated into acetone. The polymer was purified three times with acetone, followed by dialysis against distilled water for 3 days. Finally, the obtained polymer was lyophilized. The reaction yield reached 61%.

#### 4.2.2. Chitosan Particulate Hydrogels Preparation

The selected method for particle preparation was the double crosslinking (ionic and covalent) technique in a water-in-oil emulsion, the advantage being the use of a lower amount of covalent crosslinker, which leads to a decrease in CPH toxicity. The fundamental principle of the process is the formation of an interconnected/interpenetrated network in the first stage through the majority ionic crosslinking process, followed by the covalent crosslinking step. CPH-based CS-PEGA was prepared in reverse emulsion (*w/o*) according to our previous study [17]. At first, CS-PEGA was dissolved in 50 mL CH_3_COOH (1% *w*/*w*) to produce solutions with concentrations of 0.35%; 0.5%; and 0.75% wt. Then, Tween 80 was added to the solution (2% wt) and stirred vigorously for 5 min. Next, the organic phase was prepared: 200 mL toluene and Span 80 (2% wt). Aliquots (250 μL) of polymer solution were added into the organic phase, under ultra Turrax stirring (5000–15,000 rpm), leading to emulsion formation. Subsequently, freshly prepared crosslinker aqueous solutions, namely TPP (5% wt), were added according to the calculated amounts. After 10 min, the obtained emulsion was transferred to a mechanical stirring-equipped reactor, where the ionic cross-linking process continued at 500 rpm for 1 h at room temperature. Afterward, the covalent cross-linking process was carried out by adding 5 mL glutaraldehyde (GA) extracted in toluene (c = 1.12 mg/mL) for 1h. Obtained CPH were centrifuged at 5000 rpm for 1h and resuspended in acetone after removing the organic phase. The unreacted reagents (TPP, GA, respectively surfactants) were removed by successive washing with water, acetone, and hexane. The purified CPH were weighted and characterized to determine their composition (FTIR, TGA), size distribution (laser diffractometry), zeta potential, morphology (scanning electron microscopy (SEM)), swelling behavior in an aqueous environment, hemocompatibility, cytotoxicity, drug loading, and in vitro drug release.

#### 4.2.3. Characterization of Chitosan Derivatives and Micro/Nanoparticles

##### Techniques of Characterization Used

Fourier Transform Infrared Spectroscopy (FT-IR) spectra of CS, PEGA, CS-PEGA derivative, and CPH were recorded with a DIGILAB SCIMITAR FTS 2000 spectrometer. The samples were prepared as KBr pellets and scanned over the wave number range of 4000−450 cm^−1^ at a resolution of 4.0 cm^−1^.

^1^H NMR spectra were obtained using a Bruker Avance DRX 400 (Bruker, Rheinstetten, Germany). CS, PEGA (Mn = 480), and CS-PEGA derivatives were dissolved in D_2_O + HCl or D_2_O according to their solubility. The substitution degree (DS) was calculated from the peak area at the chemical shift of -CH_2_-proton against that of NHAc proton. NMR spectra were recorded in HCl/D_2_O using a 400 MHz spectrometer (experimental section).

Thermo Gravimetric Analysis (TGA) was carried out with a Mettler Toledo model TGA/SDTA 851 (TGA Q 500–1285 system). Dried CPH (5 mg) were loaded in an open aluminum pan, heated under a dynamic N_2_ atmosphere, with a heating rate of 10^0^ C/min, in a temperature range from room temperature to 800 °C.

CPH morphology was investigated by SEM technique. SEM images were recorded with a HITACHI SU 1510 (Hitachi SU-1510, Hitachi Company, Tokyo, Japan) Scanning Electron Microscope, CPH were fixed on an aluminum stub and coated with a 7 nm thick gold layer using a Cressington 108 device before observation.

CPH sizes were analyzed by laser diffractometry technique (SHIMADZU SALD 7001, Shimadzu Company, Kyoto, Japonia ). CPH (3 mg) were immersed in acetone and sonicated for 15 min at room temperature using a sonication bath (Bandelin Sonorex, Bandelin Company, Berlin, Germany). CPH suspension was added to a quartz cuvette equipped with a stirring mechanism and analyzed. All measurements were performed in triplicate.

CPH Zeta potential was analyzed with Zetasizer Nano ZS Series from Malvern (Malvern, UK). CPH were dispersed in distilled water at a mass concentration of 1.25 g/L and sonicated. The determinations were made in triplicate at 25 °C with a Zetasizer Nano Series ZS from Malvern.

#### 4.2.4. CPH Swelling Behavior in Aqueous Media

CPH swelling characteristics in an aqueous environment were evaluated by the gravimetric method using buffer solutions with different pH (slightly alkaline aqueous medium with pH 7.4 (Phosphate Buffer Solution, PBS) and acidic aqueous medium with pH 3.3 (Acetate Buffer Solution, ABS)).

0.030g of CPH were immersed in a 2 mL Eppendorf tube containing 1 mL of ABS or PBS. The suspensions were monitored and maintained at room temperature under mechanical stirring (100 rpm) for 24 h when equilibrium swelling was attained. CPH suspensions were ultra-centrifuged (15,000 rpm), withdrawn from the buffer solutions, and weighed at specified time intervals, and immediately replaced with an equivalent volume of fresh buffer. The maximum swelling degree was determined using (Equation (4)):

(4) $$Q\%=\frac{w_{s}-w_{0}}{w_{0}}\times 100$$

where w_s_ is the weight of swollen CPH; w_0_ is the weight of dry CPH.

#### 4.2.5. CPH Drug Loading Capacity

Levofloxacin (LEV) a fluoroquinolone antibacterial used as a model drug for loading into the CPH drug delivery system. The drug loading process was carried out through a diffusional mechanism. An accurately weighed amount of CPH (0.030g) was suspended in a 1 mL aqueous solution of LEV (c = 25 mg/mL) and dispersed by sonication for 15 min. The suspensions were maintained at room temperature, under gentle mechanical stirring for 72 h. After preset time intervals CPH samples were centrifuged (15,000 rpm, 5 min). The quantity of LEV encapsulated by CPH was determined by calculating the drug content from the supernatant with Equation (5) based on a calibration curve, previously prepared.

(5) $$y=6.0424\cdot x;{\;R}^{2}=0.9973$$

Drug encapsulation efficiency (DEE) represents the quantity of added drug (%) that is encapsulated in the formulation. The DEE of LEV from CPH was calculated with Equation (6). The LEV entrapment efficiency of CPH was determined with Equation (7).

(6) $$\mathrm{DEE}=\frac{\mathrm{Actual}\;\mathrm{drug}\;\left(\mathrm{LEV}\right)\mathrm{content}\;\mathrm{loaded}\;\mathrm{in}\;\mathrm{CPH}}{\mathrm{Theoretical}\;\mathrm{drug}\;\mathrm{content}\;\left(\mathrm{LEV}\right)}\times 100$$

(7) $$\mathrm{Entrapment}\;\mathrm{efficiency}=\frac{\mathrm{Total}\;\mathrm{amount}\;\mathrm{of}\;\mathrm{LEV}-\mathrm{Amount}\;\mathrm{of}\;\mathrm{LEV}\;\mathrm{from}\;\mathrm{supernatant}}{\mathrm{Total}\;\mathrm{amount}\;\mathrm{of}\;\mathrm{LEV}}\;\;$$

All tests were accomplished with a spectrophotometer UV–VIS NanoDrop ND-1000. LEV-loaded CPH were freeze-dried. The tests were made in triplicate and the results were averaged.

#### 4.2.6. In Vitro LEV Release from CPH

To evaluate the in vitro drug release property, the release of LEV-loaded CPH was performed in PBS (pH = 7.4); this environment ensures a slower release rate of the drug. The LEV release process was carried out by immersing 0.030 g of CPH in a 2 mL Eppendorf tube containing 1 mL of PBS. The suspensions were maintained at 37 °C, under continuous gentle stirring (50 rpm). After pre-established time intervals, the suspensions were ultra-centrifuged (15,000 rpm) and the drug quantity was determined by UV–Vis spectrometry at 287 nm. The determinations were made in triplicate and the results were averaged.

#### 4.2.7. CPH Hemocompatibility

Particle hemolysis tests were performed according to a method suggested by Vuddanda et al. [42] presented in our previous study [16,17]. Briefly, 2 mL of CPH were suspended in saline solution with different concentrations and mixed with purified red blood cells (RBC, 2 mL, at final concentrations of 100, 250, and 500 mg CPH/mL). Samples were incubated at 37 °C for 2, 4, and 6 h and gently shaken every 30 min for re-suspension. After a preset time, samples were centrifugated (2000 rpm, 5 min) and 1.5 mL of supernatant was incubated for another 30 min at room temperature, allowing the hemoglobin oxidation. Finally, the oxyhemoglobin from the supernatant was spectrophotometrically analyzed with a spectrophotometer (PG Instruments T60 UV-Vis Spectrophotometer) at 540 nm. Hemolysis was determined using Equation (8). The determinations were made in triplicate and the results were averaged.

(8) $$\%\;\mathrm{Hemolysis}=\frac{\mathrm{Abs}\;\mathrm{sample}-\mathrm{Abs}\;\mathrm{negativ}\;\mathrm{control}}{\mathrm{Abs}\;\mathrm{pozitiv}\;\mathrm{control}-\mathrm{Abs}\;\mathrm{negativ}\;\mathrm{control}}$$

#### 4.2.8. Cytotoxicity

##### Cell Culture

All cells were cultured in a humidified atmosphere at 37 °C with 5% CO_2_. MCF-10A cells were cultured in DMEM/F12 supplemented with 5% horse serum, 20 ng/mL EGF, 10 µg/mL insulin, 0.5 ng/mL hydrocortisone, 10 ng/mL cholera toxin, 1% Pen/Strep; HDMVEC (primary dermal microvascular endothelial cells) were cultured in Vascular Cell Basal Medium supplemented with Microvascular Endothelial Cell Growth Kit-BBE ATCC^®^ PCS-110-040.

## Figures and Tables

**Table 1:** CPH—preparation parameters and size measurements.

Sample Code	Polymer Solution Concentration, %	Molar Ratio NH_2_/Na_5_P_3_O_10_	Speed, Rpm	Water Phase, mL	The Organic Phase, mL	Surfactants Concentration, %	Ionic Crosslinking Time, min	Yields %	Average Diameter (LD), µm	Potential Zeta (mV)
A1	0.5	1:05	5000	50	200	2	60	32	Aggregates	-
A2	0.5	1:1	5000	50	200	2	60	41	Aggregates	-
A3	0.5	1:2	5000	50	200	2	20	36	Aggregates	-
A4	0.5	1:2	5000	50	200	2	40	44	Aggregates	-
A5	0.5	1:2	5000	50	200	2	60	79	3.548	15.4 ± 0.2
A6	0.5	1:2	9000	50	200	2	60	80	3.263	15.2 ± 0.1
A7	0.5	1:2	12,000	50	200	2	60	76	0.708	14.9 ± 0.2
A8	0.5	1:2	15,000	50	200	2	60	78	0.562	14.8 ± 0.2
A9	0.35	1:2	15,000	50	200	2	60	56	0.089	11.9 ± 0.2
A10	0.5	1:3	15,000	50	200	2	60	59	0.141	15.5 ± 0.1
A11	0.75	1:2	15,000	50	200	2	60	51	0.730	15.8 ± 0.1

**Table 2:** Maximum water uptake of A5–A11 samples.

Sample Code	A5	A6	A7	A8	A9	A10	A11
Q _ABS_, %	835	838	886	985	1144	995	904
Q _PBS_, %	511	541	580	584	669	559	598

**Table 3:** Encapsulated LEV and efficiency.

Sample Code	A5	A6	A7	A8	A9	A10	A11
mg LEV/mg CPH	0.63	0.69	0.62	0.75	0.67	0.48	0.66
Encapsulation efficiency, %	62.34	66.36	58.76	76.41	66.36	47.32	66.53

**Table 4:** Released drug and efficiency.

Sample Code	A5	A6	A7	A8	A9	A10	A11
mg LEV/mg CPH	0.31	0.30	0.31	0.42	0.35	0.27	0.27
Release efficiency, %	49	44	49	50	60	57	41

## Data Availability

Not applicable.

## Supplementary Materials

The following supporting information can be downloaded at: https://www.mdpi.com/article/10.3390/gels8080494/s1, Figure S1: TGA analysis of chitosan particulate hydrogels (A8, A9, A10 samples); Figure S2: Kinetics of the LEV loading into the ABS from chitosan particulate hydrogels (A8, A9 samples); Figure S3: Kinetics of the LEV release efficiency into the PBS from chitosan particulate hydrogels (A8, A9 samples); Figure S4: Korsmeyer-Peppas model for A8, 9 samples; Figure S5: Viability of HDMVEC and MCF-10A cells for submi chitosan particulate hydrogels (sample A9); Table S1: Characteristic absorption bands of CS, PEGA, CS-PEGA; Table S2: CPH characteristic absorption bands; Table S3: n and k values obtained based on the Korsmeyer-Peppas model (0–480 min).

## Abbreviations

ABS—Acetate Buffer Solution
CH3COOH—Acetic acid
CS—Chitosan
DEE—Drug Encapsulation Efficiency
DS—Degree of substitution
Eq.—Equation
FTIR—Fourier Transform Infrared Spectroscopy
GA—Glutaraldehyde
GlcN—2-amino-2-deoxy-β-d-glucopyranosyl
GlcNAc—2-acetamido-2-deoxy-β-d-glucopyranosyl
LEV—Levofloxacin
CPH—Chitosan particulate hydrogels
NMR—Nuclear Magnetic Resonance
PBS—Phosphate buffer solution
PEG—Poly (ethylene glycol)
PEGMA—Poly (ethylene glycol) methacrylate
PEGA—Poly (ethylene glycol) methyl ether acrylate
CS-PEGA–Chitosan grafted poly (ethylene glycol) methyl ether acrylate
RBC—Red blood cells
RT—Room temperature
SEM—Scanning Electron Microscope
Span 80—Nonionic surfactant
TGA—Thermo Gravimetric Analysis
Tween 80—Nonionic Surfactant
TPP—Sodium tripolyphosphate
