# Supplementary material for: Chitosan Grafted Poly (Ethylene Glycol) Methyl Ether Acrylate Particulate Hydrogels for Drug Delivery Applications
Source: Gels. 2022 Aug 9;8(8):494. doi: 10.3390/gels8080494 (PMC9407074; doi:10.3390/gels8080494)
Supplement: Supplementary file 1 [file gels-08-00494-s001.zip › gels-1832182-supplementary.pdf]

Supplementary Materials

Chitosan grafted poly(ethylene glycol) methyl ether acrylate submicronic pH sensitive hydrogels for drug delivery applications

Corina-Lenuța Savin (Logigan)[1,2], Christelle Delaite[2] , Crina Tiron[3], Cristian Peptu[4], Marcel Popa[1,5,6] , Catalina Anișoara Peptu[1],*

1 Department of Natural and Synthetic Polymers, Faculty of Chemical Engineering and Environmental Protection "Cristofor Simionescu", "Gheorghe Asachi" Technical University of Iasi, Romania, savincorina@yahoo.com, marpopa@ch.tuiasi.ro, catipeptu@ch.tuiasi.ro

2 Laboratory of Photochemistry and Macromolecular Engineering Institute J.B. Donnet, University of Haute Alsace, Mulhouse, France, christelle.delaite@uha.fr

3 Regional Institute of Oncology, 2-4, General Henri Mathias Berthelot Street, 700483 Iasi, Romania; crinatiron2003@yahoo.com

4 "Petru Poni" Institute of Macromolecular Chemistry, Aleea Grigore Ghica Voda, 41A, 700487 Iasi, Romania, cristian_peptu@yahoo.com

5 "Apollonia" University of Iasi, Faculty of Medical Dentistry, Muzicii Street, No. 2, Iasi 700511, Romania

6 Academy of Romanian Scientists, Splaiul Independentei Street, No 54, 050094 Bucharest, Romania

* Correspondence: catipeptu@ch.tuiasi.ro (Peptu C.A.)

Contents

**1.**	Characteristic absorption bands of CS, PEGA, CS-PEGA	***Table S1***	*Page 2*
**2.**	CPH characteristic absorption bands	***Table S2***	*Page 2*
**3.**	TGA analysis of chitosan particulate hydrogels (A8, A9, A10 samples)	***Figure S1***	*Page 2*
**4.**	Kinetics of the LEV loading into the ABS from chitosan particulate hydrogels (A8, A9 samples)	***Figure S2***	*Page 3*
**5.**	Kinetics of the LEV release efficiency into the PBS from chitosan particulate hydrogels (A8, A9 samples)	***Figure S3***	*Page 4*
**6.**	Korsmeyer-Peppas model for A8, 9 samples	***Figure S4***	*Page 5*
**7.**	**n** and **k** values obtained based on the Korsmeyer-Peppas model (0 – 480 min)	***Table S3***	*Page 5*
**8.**	Viability of HDMVEC and MCF-10A cells for submi chitosan particulate hydrogels (sample A9)	***Figure S5***	*Page 5*

Table S1. Characteristic absorption bands of CS, PEGA, CS-PEGA

Compound	Wavelength (cm^{-1})	Absorption band
**Chitosan**	1076	Stretching vibrations of -C-O-C
	1379,1423,1659	Specific signals for deformation vibrations of acetylated amine (NHAc) (amide I, $-NH_2$, and amide III)
	2879	The vibration of CH groups
	3355	Axial stretching vibrations of OH and NH_2 groups
**PEGA**	1105	Stretching vibrations -C-O-C
	1724	The vibration signal corresponding to the C = O bonds
	2874	The vibration of CH groups
	3522	Axial stretching vibrations of OH groups
**CS-PEGA**	1083	Stretching vibrations -C-O-C
	1379, 1566,1649	Specific signals for deformation vibrations of acetylated amine groups
	1733	The vibration signal corresponding to the C = O bonds
	2880	The vibration of CH groups
	3470	Axial stretching vibrations of secondary amine groups

Table S2. CPH characteristic absorption bands

Compound	Wavelength (cm^{-1})	Absorption band
**A8**	846	The absorption band signal corresponding to the new bonds formed by the ionic crosslinking process between $P_3O_{10}^{5-}$ anions from TPP and NH_3^+ cations from CS-PEGA
	1074	Characteristic stretching vibrations -C-O-C
	1315	Band-specific bonding signal P = O
	1573	Signals specific to imine linkages -C=N-

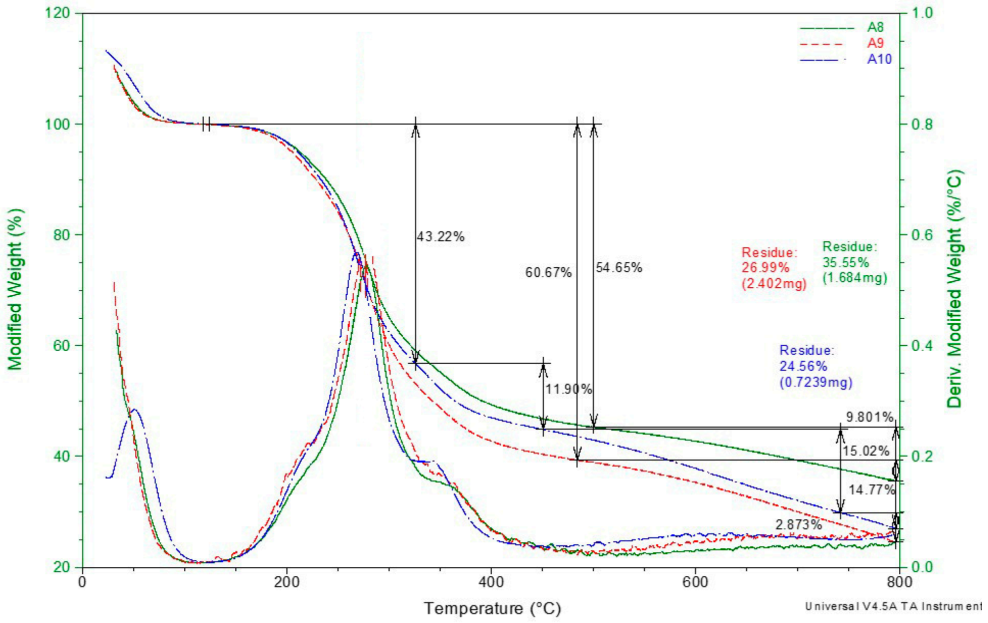

Figure S1. TGA analysis of chitosan particulate hydrogels (A8, A9, A10 samples)

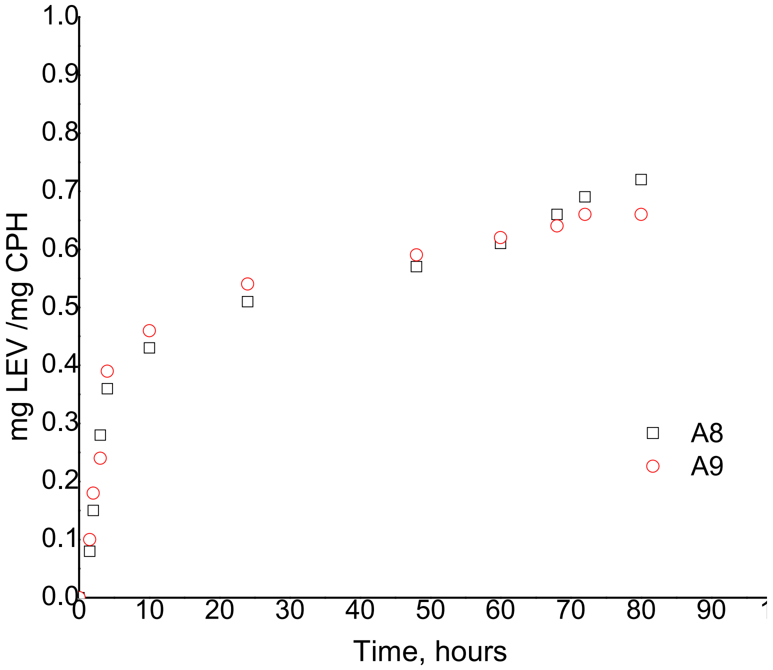

Figure S2. Kinetics of the LEV loading into the ABS from chitosan particulate hydrogels (A8 - A9 samples)

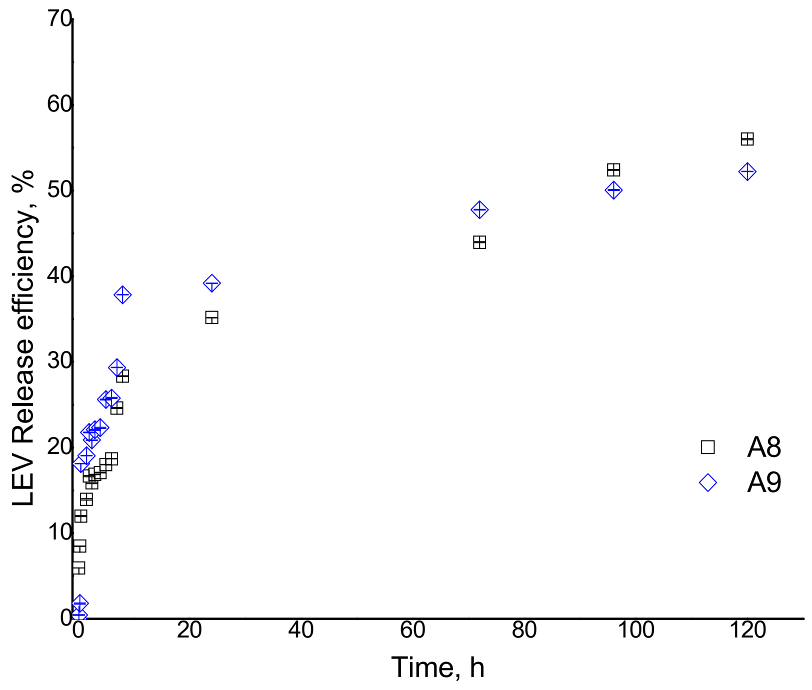

Figure S3. Kinetics of the LEV release efficiency into the PBS from chitosan particulate hydrogels (A8 - A9 samples)

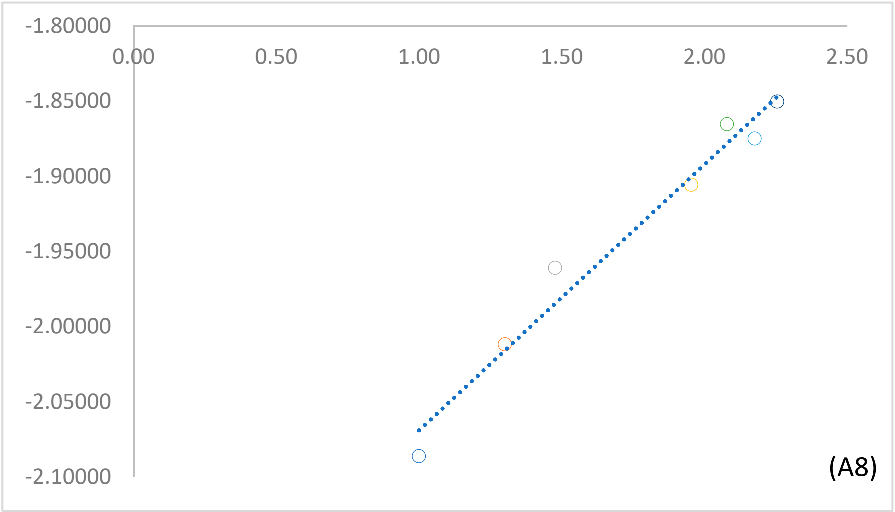

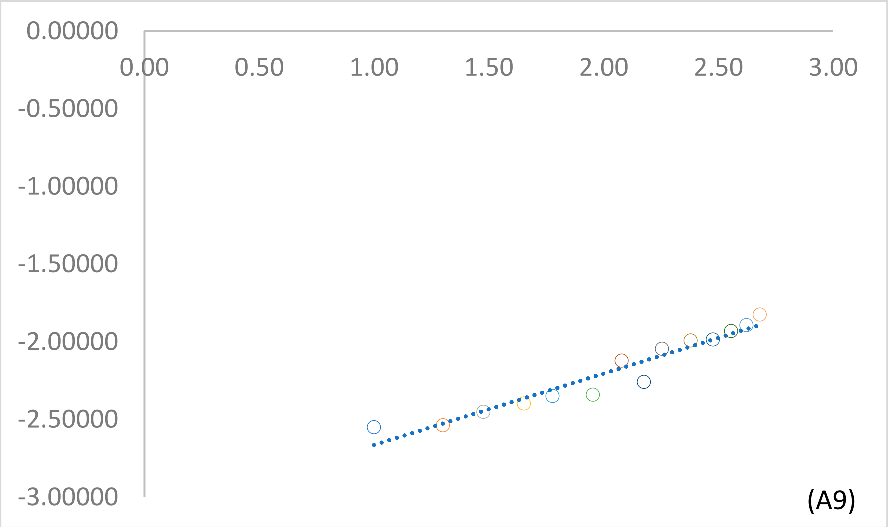

Figure S4. Korsmeyer-Peppas model for samples A8-A9

Table S3. *n* and *k* values obtained based on the Korsmeyer-Peppas model (0 – 480 min)

Sample code	Equations	k	n	R^2
**A5**	$y = 0.4593 \cdot x - 3.1908$	0.1306	0.4593	0.9277
**A6**	$y = 0.4517 \cdot x - 3.3124$	0.1327	0.4517	0.9664
**A7**	$y = 0.4018 \cdot x - 3.1908$	0.1726	0.4218	0.9318
**A8**	$y = 0.4294 \cdot x - 3.351$	0.4214	0.4294	0.9778
**A9**	$y = 0.4176 \cdot x - 2.2459$	0.2433	0.4176	0.9230
**A10**	$y = 0.4228 \cdot x - 2.2956$	0.1575	0.4228	0.9314
**A11**	$y = 0.6035 \cdot x - 31561$	0.0319	0.6035	0.9378

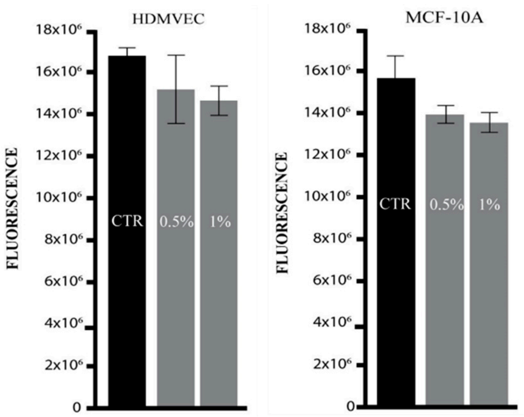

Figure S5. Viability of HDMVEC and MCF-10A cells for chitosan particulate hydrogels (sample A9)
